# Protein Environment: A Crucial Triggering Factor in Josephin Domain Aggregation: The Role of 2,2,2-Trifluoroethanol

**DOI:** 10.3390/ijms19082151

**Published:** 2018-07-24

**Authors:** Cristina Visentin, Susanna Navarro, Gianvito Grasso, Maria Elena Regonesi, Marco Agostino Deriu, Paolo Tortora, Salvador Ventura

**Affiliations:** 1 Institut de Biotecnologia i de Biomedicina and Departament de Bioquímica i Biologia Molecular, Universitat Autònoma de Barcelona, Bellaterra, 08193 Barcelona, Spain; cristina.visentin@unimi.it (C.V.); Susanna.Navarro.Cantero@uab.cat (S.N.); 2 Istituto Dalle Molle di Studi sull’Intelligenza Artificiale (IDSIA), Scuola Universitaria Professionale della Svizzera italiana (SUPSI), Università della Svizzera italiana (USI), CH-6928 Manno, Switzerland; gianvito.grasso@supsi.ch (G.G.); marco.deriu@supsi.ch (M.A.D.); 3 Dipartimento di Biotecnologie e Bioscienze, Università di Milano-Bicocca, 20126 Milano, Italy; mariaelena.regonesi@unimib.it; 4 Centro di Neuroscienze di Milano (Neuro-MI), 20126 Milano, Italy

**Keywords:** ataxin-3, josephin domain, 2,2,2-trifluoroethanol, amyloid aggregation, molecular dynamics, protein-cosolvent interaction

## Abstract

The protein ataxin-3 contains a polyglutamine stretch that triggers amyloid aggregation when it is expanded beyond a critical threshold. This results in the onset of the spinocerebellar ataxia type 3. The protein consists of the globular N-terminal Josephin domain and a disordered C-terminal tail where the polyglutamine stretch is located. Expanded ataxin-3 aggregates via a two-stage mechanism: first, Josephin domain self-association, then polyQ fibrillation. This highlights the intrinsic amyloidogenic potential of Josephin domain. Therefore, much effort has been put into investigating its aggregation mechanism(s). A key issue regards the conformational requirements for triggering amyloid aggregation, as it is believed that, generally, misfolding should precede aggregation. Here, we have assayed the effect of 2,2,2-trifluoroethanol, a co-solvent capable of stabilizing secondary structures, especially α-helices. By combining biophysical methods and molecular dynamics, we demonstrated that both secondary and tertiary JD structures are virtually unchanged in the presence of up to 5% 2,2,2-trifluoroethanol. Despite the preservation of JD structure, 1% of 2,2,2-trifluoroethanol suffices to exacerbate the intrinsic aggregation propensity of this domain, by slightly decreasing its conformational stability. These results indicate that in the case of JD, conformational fluctuations might suffice to promote a transition towards an aggregated state without the need for extensive unfolding, and highlights the important role played by the environment on the aggregation of this globular domain.

## 1. Introduction

Spinocerebellar ataxia type 3 (SCA3) is one of the nine known polyglutamine (polyQ) diseases [1]. These disorders are caused by CAG triplet repeats over the critical threshold of about 50 in the coding sequence of the respective gene. The only common feature among these disease-related proteins is the presence of a polyQ tract that triggers protein aggregation in the presence of the expansion [2,3]. The resulting intra-neuronal amyloid aggregates are the hallmark of the pathology.

Ataxin 3 (ATX3), the causative protein of SCA3, is a deubiquitinating enzyme localized to both the nucleus and cytoplasm [4]. It consists of a globular N-terminal domain, called Josephin Domain (JD), and a disordered C-terminal tail where the polyQ tract and one or two ubiquitin-interacting motifs are located [5]. SCA3 onset is triggered by variants carrying more than 55 glutamines. The aggregation process consists of two stages: The first is polyQ independent and occurs in all ATX3 variants, whereas the second is only triggered by an expanded polyQ tract and results in final amyloid-like fibrils generation [6,7,8]. However, the expanded polyQ tract does not significantly affect protein stability but enhances local structural fluctuations of the flanking region, in particular of two critical α-helices of the JD [8]. JD is known to have an intrinsic amyloidogenic potential, contributed to mainly by residues Ile77-Gln78 and Trp87, which are part of JD ubiquitin binding sites [9,10]; although recent computational studies also suggest that JD-JD binding might be mediated by other residues such as Arg101, followed by a conformational rearrangement of the complete JD without significant secondary structure changes [11,12,13]. Furthermore, experimental and computational studies focusing on the interaction between JD and other proteins [11,13], or inorganic surfaces [14], pointed to the protein environment as a crucial triggering factor for JD aggregation. 

Solvent-protein interactions strongly influence the aggregation properties of proteins [15,16]. The fluorinated alcohol 2,2,2-trifluoroethanol (TFE) is perhaps the most used cosolvent to investigate protein aggregation. The action of TFE can be accounted for by two major effects: (i) tertiary structure disruption and (ii) secondary structure stabilization, especially α-helix. The stabilized secondary structure is sequence dependent [17] and reflects the native composition [18]. The suggested molecular mechanism relies upon an increase in the free energy of the random coil state, because TFE-water mixtures are less able to solvate the amide group of the peptide backbone [19], which results in reinforced intramolecular hydrogen bonds among amide groups [20]. At high TFE concentration, the formation of partially folded states also occurs due to the disruption of hydrophobic contacts in the protein core and this feature is usually exploited to promote protein aggregation [21,22]; however, in particular cases, the interactions between TFE and the protein instead protect it against aggregation [23].

Here we report a combined experimental and computational study aimed at characterizing the effects of TFE on JD conformation, stability and aggregation propensity. For this purpose, circular dichroism (CD) was employed to evaluate JD secondary structure changes at different TFE concentrations; spectroscopic analyses provided information on TFE effects before and after 24 h of co-incubation; transmission electron microscopy (TEM) and Fourier transform infrared (FTIR) spectroscopy, informed about the morphological and structural properties of JD aggregates, formed in the presence and the absence of TFE, and chemical and thermal denaturations on the impact of this co-solvent on JD stability. Molecular modeling was employed to investigate how TFE may affect secondary structure and conformational arrangement of the JD. Moreover, from the atomic resolution trajectories, residues most likely involved in JD-TFE binding were identified.

Thanks to the above-mentioned multidisciplinary approach, this work sheds light on the role of the protein environment on amyloidogenesis by exploring how TFE-JD interactions lead to JD aggregation prior to protein misfolding.

## 2. Results

### 2.1. TFE Increases the α-Helix Content of the Josephin Domain

**Figure 1:**
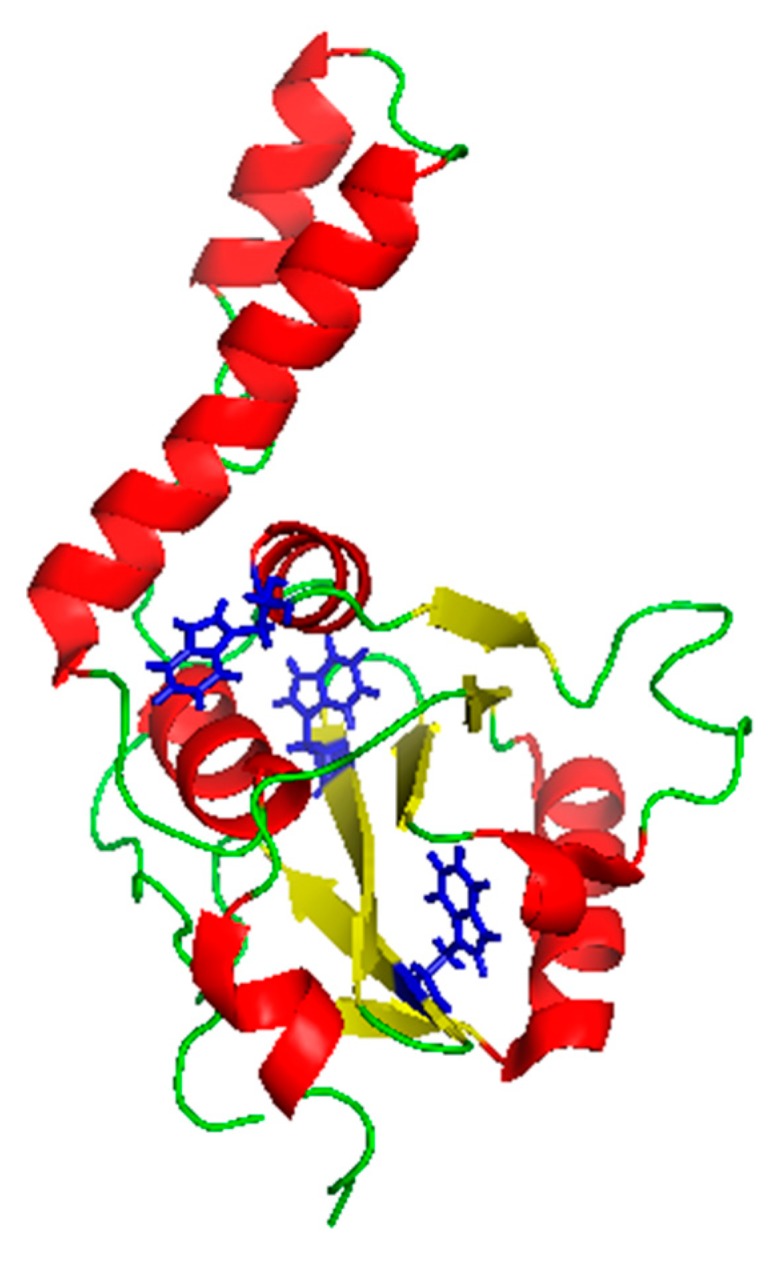
Ribbon representation of the JD structure. The Protein Data Bank accession code for the structure is 1ZYB with tryptophan residues highlighted in blue. The figure was prepared with PyMOL. Red: Helices; yellow: beta-strands; green: disordered stretches.

TFE is known for its capability to stabilize secondary structures, preferentially α-helices, whereas hydrophobic interactions are stabilized only at the lowest concentrations and impaired at the highest [24]. Recently, it has been demonstrated that the aggregation of expanded ATX3 starts from a critical α-helix located in the JD structure (presented in Figure 1) that turns into a β-sheet after an aberrant interaction with other ATX3 monomers. For this reason, we decided to assess whether TFE is capable to modulate ATX3 aggregation via α-helix stabilization.

**Figure 2:**
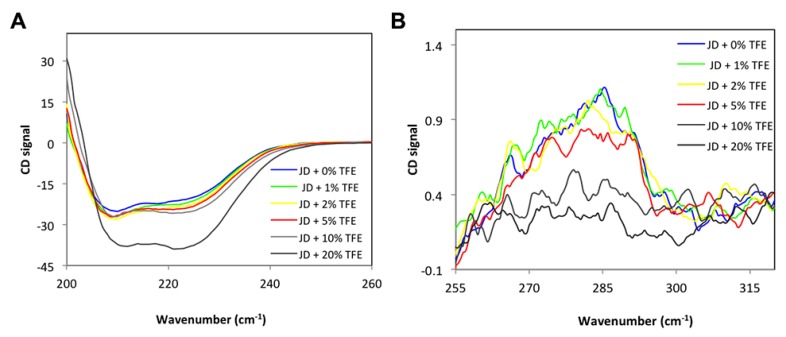
CD spectra of JD at different TFE concentrations (*v*/*v*). (**A**) Far UV CD spectra; (**B**) near UV CD spectra. All measurements were performed at 25 °C.

To monitor the effects of TFE on JD structure, we recorded CD spectra in the far and near UV (Figure 2A,B) in the presence of 0%, 1%, 2%, 5%, 10%, 20% TFE. As reported in Figure 2A, the untreated JD structure is mainly α-helical although it also displays a significant β-sheet content, in keeping with a previous report [25]. After TFE addition, we observed an increase in the α-helix signal. However, although the α-helical content increased with increasing TFE, the changes in the CD spectra were rather moderate up to 10% of the co-solvent, resembling those of the untreated protein. In the near UV, the signal decreased with increasing TFE, but the CD spectra remained similar to that of the untreated sample up to 5% of the co-solvent (Figure 2B). The near UV signal was severely affected in the presence of 10% and 20% TFE, indicating significant tertiary structure loss; therefore, we decided to perform all the subsequent experiments using TFE in the range 0–5%, since our aim was to study the aggregation of JD starting from native-like conformations.

**Figure 3:**
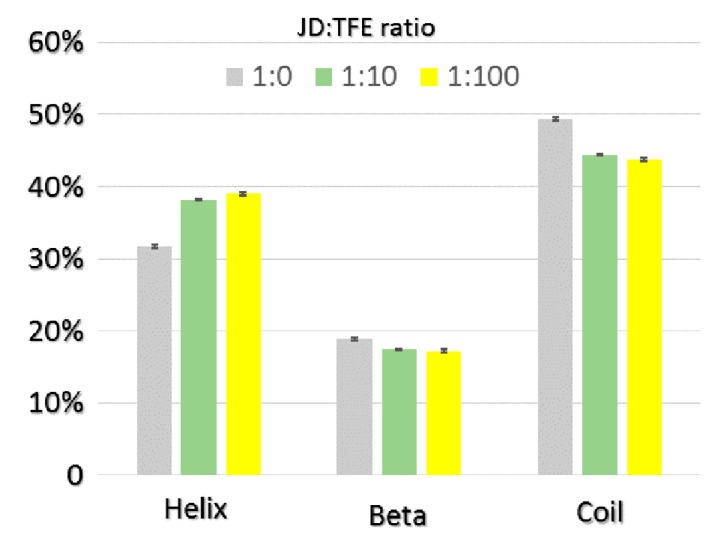
JD secondary structure percentage calculated over the last 20 ns of each MD trajectory at JD:TFE ratios of 1:0, 1:10, 1:100. The standard error is also reported.

**Figure 4:**
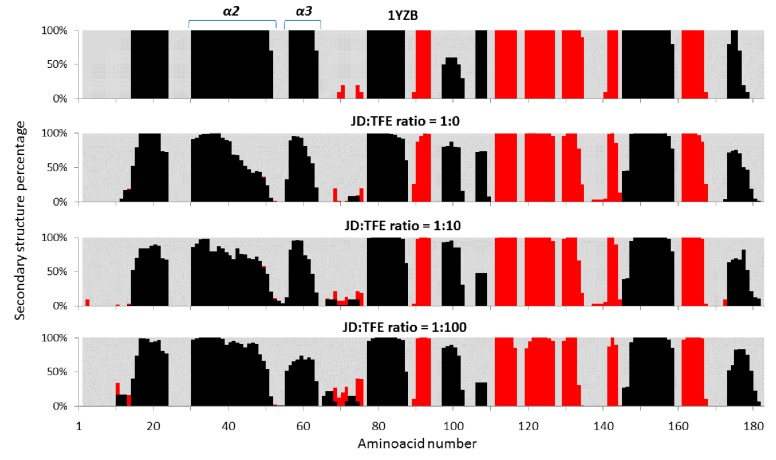
Secondary structure percentage, calculated over the last 20 ns of each MD trajectory at JD:TFE ratios of 1:0, 1:10, 1:100. MD data coming from the simulated trajectories are used as an ensemble for each concentration of TFE. The α-helix (**black**), beta-sheet (**red**) and unstructured coil (**light grey**) are represented.

The impact of TFE on JD structure was also assessed using molecular dynamics (MD). Three different JD-TFE ratios were considered: 1:0 (JD-TFE_1:0_); 1:10 (JD-TFE_1:10_), which reproduces the experimental concentration of 5% TFE; and 1:100 (JD-TFE_1:100_), reproducing an excess of TFE in solution. Outcomes from MD highlight a significant effect of TFE in modifying secondary structures of JD towards a higher α-helix content, along with a reduction in unstructured coils (Figure 3). Analysis of MD equilibrium trajectories indicated an α-helix content equal to 31% for the JD-TFE_1:0_ system, 37% for a JD-TFE_1:10_ and 39% in JD-TFE_1:100_. Secondary Structure Probability plots provide the ability to focus the attention on protein dynamics so as to investigate local protein areas responsible for the above-mentioned secondary structure changes. Figure 4 shows the JD secondary structure probability calculated over the last 20 ns of each MD trajectory. The secondary structure was proven to be reasonably conserved in JD-TFE_1:0_, with the exception of α2 and slightly in α3, in agreement with data reported earlier in the literature (11–13). In the case of JD-TFE_1:10_, a significant increase in α-helix content was detected and localized in α2. In the case of JD-TFE_1:100_, a significant modification of the secondary structure of helix α3 towards an unstructured coil and a stabilized α2 helix was instead observed. Therefore, the local JD structural modifications depend on the JD-TFE ratio. α3 structural modifications have shown to be highly correlated with JD tertiary structure arrangement (11–13); therefore, an excess of TFE is predicted to influence more profoundly JD tertiary structure, in agreement with the experimental observations. It is interesting to notice that MD simulations of this work did not highlight a direct influence of TFE on α4 conformation. Although α4 has been identified as an important aggregation-prone region, our recent computational work suggests that the α4 hydrophobic patch, Leu84-Trp87, may undergo conformational changes and exposure to the solvent when triggered by JD-JD binding [11], due to a non-local correlation with residues Arg101–Arg103.

### 2.2. Low TFE Concentrations Do Not Significantly Alter JD Structure

**Figure 5:**
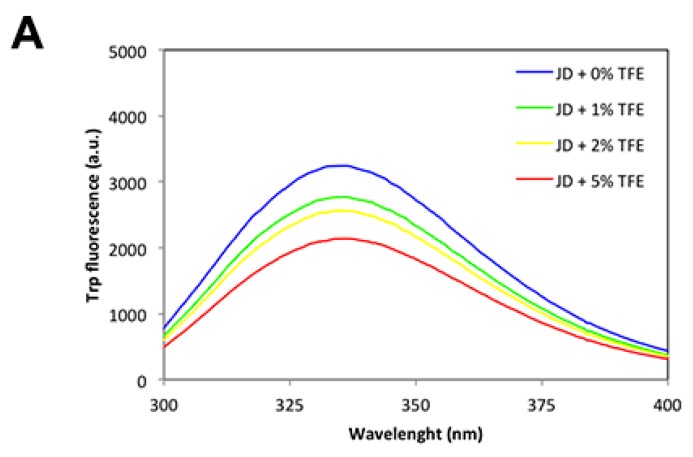

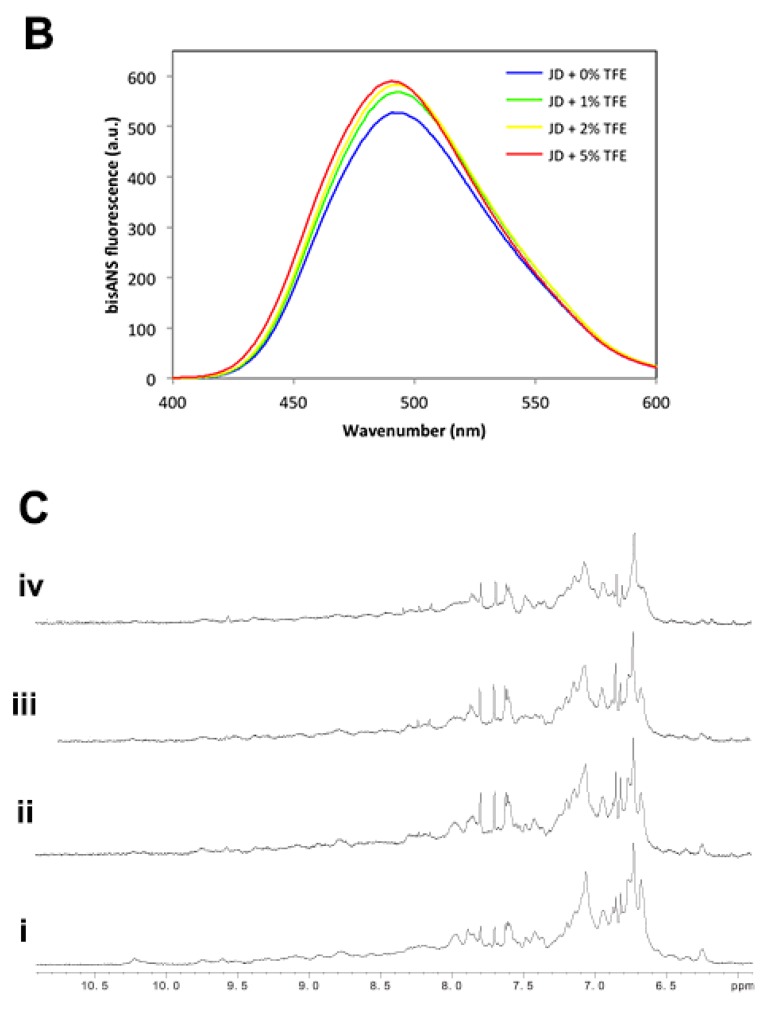
JD spectral properties immediately after TFE addition. (**A**) Tryptophan intrinsic fluorescence of freshly purified JD in the presence of 0%, 1%, 2% and 5% TFE (*v*/*v*); (**B**) BisANS binding assays of freshly prepared JD after the addition of 0%, 1%, 2% and 5% TFE (*v*/*v*); (**C**) ^1^H-NMR titration. Spectra recorded in solution containing 0% (i), 1 (ii), 2 (iii) and 5 (iv) % TFE. All the measurements were performed at 25 °C.

To better understand the effect of TFE on protein tertiary structure, we monitored Trp fluorescence after the addition of co-solvent at 0%, 1%, 2%, 5% concentrations to a solution of 80 µM freshly purified JD. As reported in Figure 5A, there was a concentration-dependent decrease of Trp spectral maxima; however, the maximum did not shift significantly to lower wavelengths, indicating that JD-Trp residues are located in a similar environment. Further characterization of the exposure of hydrophobic clusters to the solvent in the presence of TFE by monitoring the changes in 4,4′-Dianilino-1,1′-Binaphthyl-5,5′-Disulfonic Acid (bisANS) fluorescence confirms that the co-solvent does not promote a significant opening of the globular structure (Figure 5B). Consistent with these results, the one-dimensional NMR (^1^H-NMR) spectra of the JD in the presence of TFE up to 5% displayed a wide signal dispersion of resonances with good peak sharpness, characteristic of folded molecules (Figure 5C).

### 2.3. JD-TFE Binding Sites

**Figure 6:**
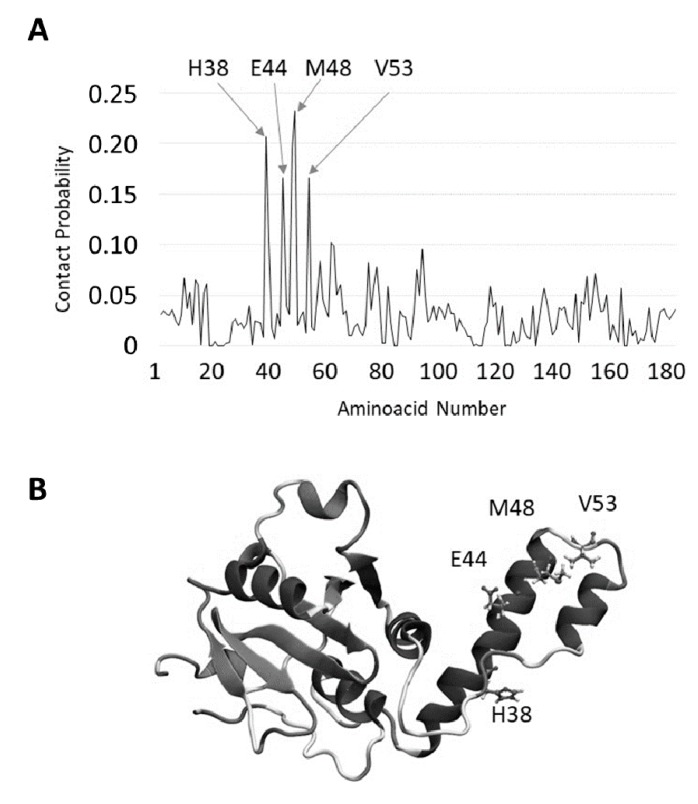
Searching for TFE-interacting residues of JD. (**A**) Most sampled TFE-JD binding regions identified by contact probability plot; (**B**) Picture of the most likely involved residues.

The most likely TFE-binding sites on the JD have been identified by MD trajectories. The lowest TFE concentration, which was shown to largely preserve JD tertiary structure, was considered for this analysis. It is interesting to notice that TFE binding sites are located in the JD hairpin domain. Major contacts (Figure 6) are located on α2 and in the connection loop between α2–α3. Noteworthy, α2 and α3 are also the regions in JD that suffer larger secondary structure modifications (Figure 4) in the presence of TFE, according to MD.

It is also worth noting that TFE behaves as a solvent that dynamically binds to and detaches from the JD structure, due to the low affinity, corresponding to a binding free energy comparable with thermal fluctuations (Supplementary Movie). Therefore, the contact probability plots are, in this case, meaningful since they provide information on the most sampled TFE-JD binding area (Figure 6).

### 2.4. TFE Promotes JD Aggregation

**Figure 7:**
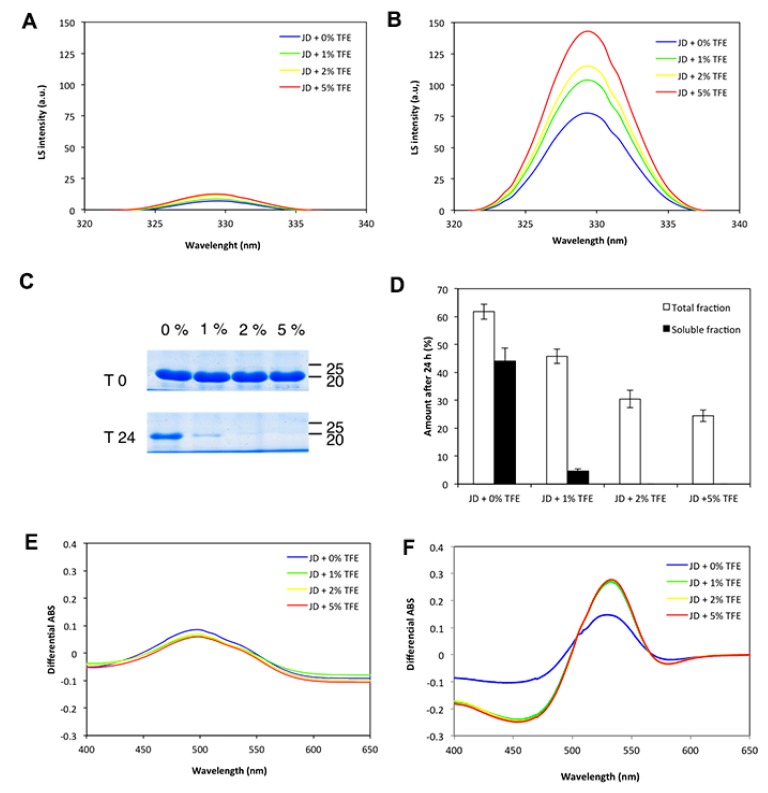
TFE-induced aggregation of JD after a 24-h incubation at 37 °C at the indicated concentrations of the osmolyte (*v*/*v*), as assessed by different spectral measurements. Light scattering of 80 µM freshly prepared (**A**) JD and after 24 h of incubation (**B**) in the presence of 0%, 1%, 2% and 5% TFE (*v*/*v*); (**C**) SDS-PAGE of the soluble fraction of JD incubated for 24 h in the presence of 0%, 1%, 2% and 5% (*v*/*v*) TFE. Gels were stained using the BluSafe Reagent; (**D**) densitometric analysis of SDS-soluble band of total and soluble fraction. Bars represent standard deviations. Congo red binding assay of freshly prepared (**E**) JD and after 24 h of incubation (**F**) in the presence of 0%, 1%, 2% and 5% TFE (*v*/*v*). Data are represented as differential spectra relative to 0 time. All the measurements were performed at 25 °C.

To monitor if TFE influences the intrinsic aggregation propensity of JD, solutions of 80 µM freshly purified JD in the presence of 0%, 1%, 2% and 5% TFE were incubated for 0 and 24 h at 37 °C under static conditions. We first monitored the presence of aggregates in fresh and incubated samples by measuring orthogonal light scattering (Figure 7A,B). A large increase in light scattering signal was observed upon incubation under all conditions: In particular, the amount of aggregates in the solution were directly related to TFE concentration, the higher amount being detected at 5% TFE. An aliquot of each incubated sample was centrifuged to separate the soluble fraction, which was then subjected to SDS-PAGE and densitometric analysis. This revealed that after 24 h, about 44% of protein in the untreated sample was still soluble, but only 5% remained soluble at 1% TFE and none at 2% and 5% TFE (Figure 7C,D).

Next, we monitored the ability of the different solutions to bind Congo red (CR), a dye typically used to detect amyloid assemblies by monitoring the typical signal shift at 540 nm upon binding. In fresh samples, the binding to CR was low and no differences between solution conditions were observed. In contrast, and in excellent agreement with the SDS-PAGE analysis, upon incubation, the protein bound more strongly to CR in the presence of water-TFE mixture than it did in pure water (Figure 7E,F).

**Figure 8:**
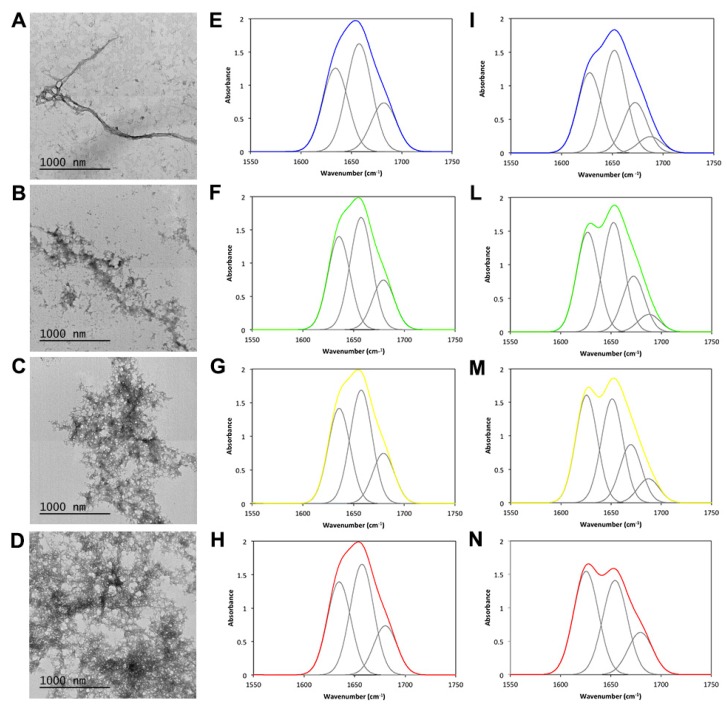
TEM and FTIR analyses of the JD aggregates in the presence of different TFE concentrations. (**A**–**D**) TEM images of negatively stained JD aggregates formed after 24 h of incubation at 37 °C in the presence of 0 (**A**), 1 (**B**), 2 (**C**) and 5% (**D**) TFE (*v*/*v*). (**E**–**I**,**L**–**N**) ATR/FTIR spectra of JD after 0 h (**E**–**H**) and 24 h (**I,L**–**N**) in the presence of 0%, 1%, 2% and 5% TFE (*v*/*v*). All the spectra were acquired in the amide I region and the fitted individual bands after Gaussian deconvolution are shown (**grey lines**).

We then proceeded with the morphological analysis of the aggregates formed after 24 h of incubation by TEM (Figure 8A–D). Clearly, on increasing TFE concentration, a strong increase in aggregated material ensued. In the presence of TFE, aggregated JD tended to cluster together forming large meshes. We next analyzed the structural features of these aggregates using FTIR and recording the amide I region of the spectra, corresponding to the absorption of the carbonyl peptide bond. Measurements were performed in the attenuated total reflection (ATR) mode, which required solvent evaporation, including TFE. After spectra deconvolution, it was possible to quantify peaks corresponding to each secondary structure, thus calculating their relative contribution to the absorbance signal (Figure 8E–I,L–N and Table S1). We compared the spectra of fresh and incubated samples. Under all conditions, the peak at 1657 cm^−1^, corresponding to α-helix, dominated the spectrum followed by a peak at 1635 cm^−1^ assigned to the native β-sheet. At zero time, all samples exhibited a similar secondary structure content. However, upon incubation, all samples experienced similar structural changes, with the β-sheet signal shifting towards 1628 cm^−1^ and a novel signal appearing at 1687 cm^−1^. These signatures are consistent with the formation of novel intermolecular and antiparallel amyloid-like β-sheets.

**Figure 9:**
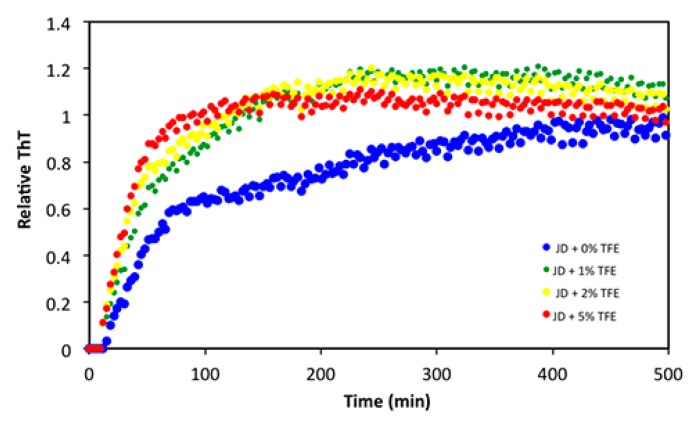
Aggregation kinetics of 40 µM JD in the presence of different TFE concentrations (*v*/*v*). The aggregation was monitored following the ThT fluorescence signal at 37 °C for 500 min (λex: 445 nm; λem: 535 nm).

Finally, we assessed whether TFE also affected the protein aggregation rate, irrespective of its impact on the amount of final aggregates. The aggregation kinetics of 40 µM JD in the presence of 0%, 1%, 2% and 5% TFE at 37 °C were recorded by monitoring changes in the fluorescent signal of Thioflavin T (ThT), a dye that increases its fluorescence when it binds to intermolecular β-sheets. As reported in Figure 9, TFE accelerated the aggregation reaction in a concentration-dependent manner.

### 2.5. TFE Impacts on JD Thermal Stability

**Figure 10:**
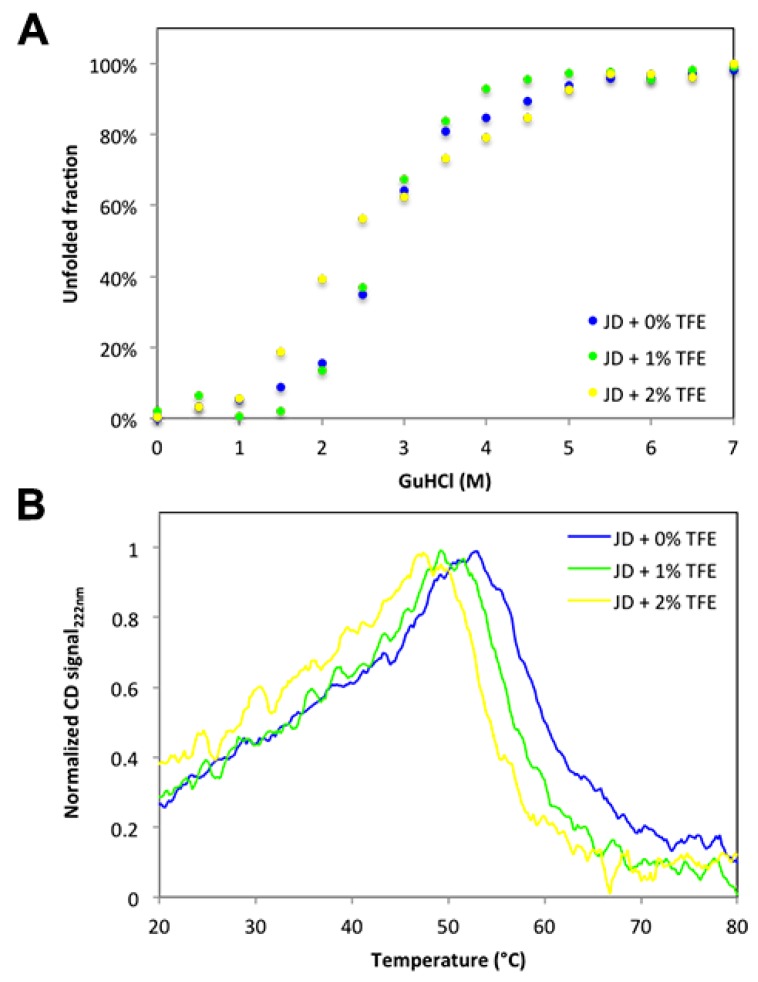

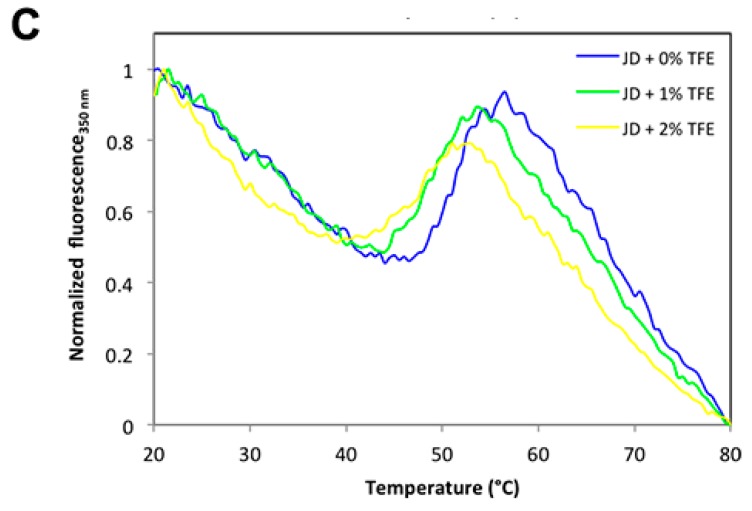
Chemical and thermal stability of JD in the presence of different TFE concentrations. (**A**) Equilibrium unfolding curves induced by GuHCl in the presence of 0%, 1% and 2% of TFE (*v*/*v*). Fluorescence emission were recorded at 25 °C and emission signal at 360 nm were plotted as function of denaturant concentration. (**B**) Thermal unfolding monitored by far-UV CD at 222 nm. Profiles were recorded at a rate of 1 °C/min in the presence of 0%, 1%, 2% and 5% of TFE (*v*/*v*). (**C**) Thermal unfolding monitored by fluorescence recording the tryptophan signal at 350 nm at a rate of 1 °C/min in the presence of 0%, 1%, 2% and 5% of TFE (*v*/*v*).

We have shown that concentrations as low as 2% TFE suffice to dramatically increase the aggregation propensity of JD, even though they impact only marginally on its structure. A possibility is that TFE would act by decreasing the conformational stability of the globular domain. Therefore, we analyzed the resistance of JD against chemical denaturation in the presence of 0%, 1%, and 2% TFE. The chemical denaturation curves at equilibrium were followed at 25 °C by monitoring the changes in Trp intrinsic fluorescence at 360 nm (Figure 10A) at increasing guanidinium chloride (GuHCl) concentrations. The JD displayed a single observable transition under all conditions. We could not find any significant destabilization in the presence of TFE, with [GuHCl]_50%_ values of 2.44 M, 2.56 M and 2.35 M in the absence and in the presence of 1% and 2% TFE, respectively. Yet, it can be that this co-solvent might still impact the thermal stability of the protein. We thus monitored JD thermal unfolding by far UV CD at 222 nm and Trp intrinsic fluorescence at 350 nm (Figure 10B,C). We followed the unfolding in the temperature range 20–80 °C, with a heating rate of 1 °C/min. When monitoring far UV signal of JD in PBS, we observed a conformational transition between 45 and 65 °C with a melting temperature (T_m_) of approximately 60 °C. TFE promoted a drop in T_m_ by about 4 °C at 1% TFE and 6 °C at 2% (Figure 10B). When monitoring Trp fluorescence, the main transition in the absence of TFE occurred between 45 and 55 °C, with a T_m_ of approximately 50 °C. This indicates that the loss of secondary and tertiary structure are uncoupled, the tertiary structure being lost first. Indeed, at 60 °C all the tertiary structure is lost whereas the protein still displays 50% of secondary structure. The presence of 1% or 2% TFE only slightly decreases the T_m_, i.e., to about 47 and 45 °C, respectively (Figure 10C). Thus, TFE only marginally affects JD thermal stability. Importantly, aggregation reactions were monitored at 37 °C, a condition that in all cases is paralleled by the pre-transition state. This further confirms that JD is properly folded in the presence of 1% and 2% TFE.

## 3. Discussion

In the treatment of amyloid-related neurodegenerative diseases, the prevention of protein aggregation represents one feasible strategy. ATX3 is a polyQ-containing, aggregation-prone protein that triggers SCA3 when its polyQ stretch exceeds a critical threshold [1]. Its aggregation process consists of two stages: The first is polyQ independent and leads to proto-fibrillar aggregates, whereas in the second the polyQ tract drives the formation of final amyloid aggregates [10,26]. Recently, a major role in this process has been assigned to a specific α-helix of the JD (α4) that is converted into β-sheet after aberrant interactions with other JD monomers, irrespective of the presence of a polyQ stretch [10]. Earlier, our computational investigation [11] suggested that, α4, and, in particular the hydrophobic region Leu84-Trp87, may undergo conformational changes and exposition to the solvent as a consequence of the JD-JD binding.

To check whether the helical stability might modulate the aggregation of ATX3, we assessed the effect of TFE on the JD, exploiting the well-known capability of this co-solvent to stabilize helical protein regions [17,18,23,24,27]. We used JD in isolation just because the aggregation starts from this ATX3 domain, also determining the shape and structure of the final fibrils [28]. The TFE concentrations we employed here were no greater than 5%, which is generally half the concentration employed in other protein aggregation studies [23,29,30,31]. These working concentrations were chosen taking into account that at 10% and 20% TFE the CD spectra revealed a loss in tertiary structure, although they also detected an increase in α-helix signal, which is consistent with the mechanism action of TFE [32,33]. All the subsequent analyses were therefore performed at 1%, 2% and 5% TFE, so as to select conditions whereby it might stabilize α-helices while preserving the tertiary structure. This is especially true in the presence of 1% and 2% TFE, where intrinsic and bisANS fluorescence, as well as near and far CD, ^1^H-NMR spectra and thermal denaturation data, all converge to indicate that TFE promotes the population of a native-like state largely indistinguishable from the original native conformation. Similar effects were previously observed in the case of acylphosphatase [29,30].

The molecular modeling experiments devised to analyze JD-TFE interactions indicated that the interactions between the protein and the co-solvent are weak and transient in such a way that the effects of TFE on protein conformation and dynamics are mediated by its influence on the chemical-physical properties of the aqueous solvent; however, our experimental data indicate that this local stabilization does not prevent or impairs the capability of JD to undergo aggregation.

Surprisingly, the presence of 1% TFE sufficed to drastically increase both the aggregation kinetics of JD and the final amount of aggregated amyloid-like material, as monitored by light scattering, CR and ThT binding, SDS and TEM. This co-solvent concentration is one order of magnitude lower than the one used in most aggregation studies [21,22], and to the best of our knowledge, the lower TFE concentration has shown to impact the aggregation behavior of a globular protein. This reflects the exquisite sensitivity of JD aggregation to the environment.

Overall, our data are in agreement with those by Ruggeri and coworkers [34], who reported that ATX3 aggregation conforms to a novel, recently described pathway of amyloid aggregation, whereby aggregation precedes misfolding [35]. According to the model, globular protein aggregation starts from a native-like state without the need for crossing the high-energy barrier associated with misfolding events. The observed JD pro-aggregational effect of very low TFE concentrations can be explained in this context. In these solution conditions, the global protein conformation is maintained; still, the structure is slightly destabilized. This would allow larger/more frequent thermal fluctuations and thus the sampling of new native-like states with increased aggregation propensity, without a need for major unfolding events. Our results immediately suggest that drugs able to bind to the JD in ATX3, stabilizing this globular domain and minimizing its thermal fluctuations, might represent promising compounds to fight SCA3.

## 4. Materials and Methods

### 4.1. Josephin Domain Purification

The JD-encoding gene was previously cloned in a pET21-a vector and the protein was expressed in *Escherichia coli* BL21 Tuner (DE3) pLacI (*E. coli* B F ompT hsdSB (rB mB) gal dcm lacY1(DE3) pLacI (CamR); Novagen, Darmstadt, Germany) as a His-tagged protein (16). Cells were grown at 37 °C in Luria Bertani—ampicillin medium and protein expression was induced adding 0.1 mM isopropyl-β-d-1-thiogalattopyranoside at OD_600_ 0.8 for 3 h at 30 °C. Cells were resuspended in 5 mL/g wet weight of lysis buffer (25 mM potassium phosphate, pH 7.2, 150 mM NaCl, 0.5 mM phenylmethanesulfonyl fluoride, 10 mM imidazole, 10% glycerol, 1 mM 2-mercaptoethanol, 2 µg/mL aprotinin, 5 mM benzamidine, 1 µg/mL pepstatin A), incubated for 30 min at 4 °C under shaking and further sonicated. After DNase I (0.2 mg/g of cells, wet weight) addition, cell suspension was incubated 30 min at room temperature, then centrifuged 45 min at 20,000× *g*. His-tagged JD was purified with a HisTrap FF Column (GE Healthcare, Chicago, IL, USA) previously equilibrated in washing buffer (25 mM potassium phosphate, pH 7.2, 150 mM NaCl, 2 mM phenylmethanesulfonyl fluoride, 10 mM imidazole, 10% glycerol, 1 mM 2-mercaptoethanol). Before injection, the sample was filtered through a 0.45 µM pore membrane. Protein was eluted with elution buffer (25 mM potassium phosphate, pH 7.2, 150 mM NaCl, 2 mM phenylmethanesulfonyl fluoride, 150 mM imidazole, 10% glycerol, 1 mM 2-mercaptoethanol), then subjected to Sephadex G-25 PD10 desalting column previously equilibrated with PBS (25 mM potassium phosphate, pH 7.2, 150 mM NaCl). Protein concentration was determined by UV absorption, using an extinction coefficient at 280 nm of 24,750 M^−1^·cm^−1^.

### 4.2. Josephin Domain Aggregation and Soluble Fraction Analysis

Freshly purified JD was diluted to a final concentration of 80 µM in PBS in the presence of 0%, 1%, 2%, 5% TFE (*v*/*v*) and incubated 24 h at 37 °C in static conditions. For soluble fraction analysis, 15 µL of fresh and incubated protein was centrifuged for 15 min at 14,000× *g* and 3 µL of the supernatant was subjected to SDS-PAGE. For total fraction analysis, 3 µL of the protein mixtures were subjected to SDS-PAGE before centrifugation. The gels were stained with BluSafe^®^ (Thermo Fischer scientific, Waltham, MA, USA) for 30 min at room temperature. 

### 4.3. Spectroscopic Methods

CD measurements were performed in PBS at 25 °C using quarz cuvettes with 1 mm path length on a Jasco-810 spectropolarimeter equipped with a PTC-348 Peltier temperature-control system (Jasco, Easton, MD, USA). Protein concentration was 15 µM for far UV and 60 µM for near UV. Thermal unfolding was monitored by collecting the CD signal at 222 nm at temperatures increasing from 20 °C to 80 °C at 1 °C/min. Fluorescence measurement was performed in PBS at 25 °C using a 1 cm path length quartz cuvette on a Jasco FP-8200 spectrofluorometer equipped with a PTC-348 Peltier temperature-control system. Protein spectra were recorded by exciting the sample at 280 nm and detecting the emission in the range 300 to 400 nm. Each trace was the average of three accumulated spectra. Thermal unfolding was performed monitoring the signal at 350 nm and increasing the temperature from 20 °C to 80 °C at 1 °C/min. JD concentration was 20 µM. Light scattering of fresh and preincubated JD was performed by exciting the sample at 330 nm and recording the emission from 320 to 340 nm. Each trace was the average of three accumulated spectra at 25 °C.

### 4.4. Congo Red Binding

JD incubated for 24 h at 37 °C in the presence of 0%, 1%, 2%, 5% TFE (*v*/*v*) was diluted at 20 µM in PBS in the presence of 20 µM CR. Optical absorption spectra were recorded from 400 to 700 nm at room temperature on a Specord^®^ 200 plus (Analytik Jena, Jena, Germany). Spectra of protein and CR alone were recorded to subtract protein scattering and dye contribution.

### 4.5. bis-ANS Binding

Fresh and incubated JD was prepared at a final concentration of 80 µM in PBS in the presence of 0%, 1%, 2%, 5% TFE (*v*/*v*) and 25 µM 4,4′-Dianilino-1,1′-binaphthyl-5,5′-disulfonic acid dipotassium salt (bis-ANS) was added. Samples were excited at 370 nm and spectra were recorded following the emission from 400 to 600 nm at 25 °C in a Jasco FP-8200 spectrofluorometer equipped with a PTC-348 Peltier temperature-control system. A 1 cm path length quartz cuvette was used. Each trace was the average of the three spectra.

### 4.6. Aggregation Kinetics

Freshly purified JD was prepared at 80 µM in PBS in the presence of 0%, 1%, 2%, 5% TFE (*v*/*v*) and ThT was added at a final concentration of 25 µM. 200 µL of reaction mixture was loaded in 96-well plate and followed for 500 min at 37 °C in a Victor TM3 multilabel plate reader (Perkin Elmer, Waltham, MA, USA). Each reaction was prepared in triplicate. The signal at 535 nm, after excitation at 445 nm, was recorded every 3 min.

### 4.7. Fourier Transform Infrared Spectroscopy

Five (5) µL of fresh and aggregated JD at 80 µM in PBS in the presence of 0%, 1%, 2% and 5% TFE (*v*/*v*) was deposited on the diamond element of a Bruker Tensor 27 FTIR spectrometer (Bruker, Billerica, MA, USA). Measurements were performed in ATR mode after solvent evaporation. Data are reported as absorbance profile and fitted with a Gaussian distribution.

### 4.8. Transmission Electron Microscopy

For TEM analyses, aggregated JD in the presence of 0%, 1%, 2%, 5% TFE (*v*/*v*) was diluted to a final concentration of 10 µM in PBS. 10 µL of sample was deposited on a carbon-coated copper grid and incubated for 5 min at room temperature. The grid was than washed with distilled water, stained 1 min with 2% uranyl acetate (*v*/*v*) and analyzed with a H-7000 transmission electron microscope (HITACHI, Tokyo, Japan) operating at an accelerating voltage of 75 kV. 

### 4.9. Molecular Dynamics

The 1YZB model [25,36], determined by NMR technique and extensively validated in literature [25,36], was considered for the present work. Moreover, the 1YZB has been considered as the starting structure in all previous computational investigations focused on the JD of ATX3 [11,12,14,37,38]. AMBER99-ILDN force-field [39,40,41] was chosen to describe protein topology. The General Amber Force Field, GAFF, [42,43] and TIP3P model [44] were employed for TFE and water molecules, respectively. Partial charges of TFE were obtained using the AM1-BCC method [45,46]. Three different systems were considered: JD alone in water, a system composed by 1 JD and 10 TFE dispersed in water solution (JD:TFE ratio 1:10, reproducing the experimental concentration of TFE = 5% *v*/*v*), and a system composed by 1 JD and 100 TFE dispersed in water solution (JD:TFE ratio 1:100, reproducing an excess of TFE in solution). Each molecular system was solvated in a cubic box of 8 × 8 × 8 nm^3^, resulting in a molecular system of about 50,000 interacting particles. The neutralized system was first minimized by 1000 steps of the steepest descent energy minimization algorithm. Then, to increase the statistics of MD data, five replicas, differing in initial atom velocities, were created from each minimized system. Two preliminary position restraint MD simulations of 100 ps were carried out in NVT and NPT ensemble, respectively, where the heavy atoms of the proteins were restrained using a force constant of 1000 kJ·mol^−1^·nm^−2^. During the first restrained MD simulation, involving 100 ps of MD in the NVT ensemble, protein and non-protein atoms were coupled separately to temperature baths using v-rescale coupling algorithm [47] with a coupling time of 1 ps. Subsequently, the second restrained MD simulation of 100 ps was performed by keeping the pressure at 1 bar by applying Berendsen’s weak coupling method [48] with a time constant of 5 ps. Finally, the production MD was performed in NPT ensemble for 200 ns. The v-rescale algorithm [47] was again used to maintain the system’s temperature at 300 K (τT = 0.1 ps) and the pressure was maintained at 1 bar using the Parrinello-Rahman [49,50] barostat (τT = 2 ps) in the isobaric-isochoric ensemble with long-range dispersion correction applied for both the energy and pressure terms. Electrostatic interactions were calculated at every step with the Particle-Mesh Ewald method with a short-range electrostatic interaction cut off of 1.0 nm. A cut-off of 1.0 nm was also applied to Lennard-Jones interactions. The LINCS algorithm [51] approach allowed an integration time step of 2 fs. The Visual Molecular Dynamics (VMD) [52] package was used for the visual inspection of the simulated systems. GROMACS 4.6 package was used for all MD simulations and data analysis [53]. The JD residues mainly responsible for the interaction with TFE have been identified by contact probability plots [11]. The secondary structure of the protein has been calculated by the STRIDE software [54] on several snapshots along the simulation time at the conformational equilibrium, as done in previous studies [11,12,13,55,56]. Two descriptors were considered to identify the JD conformational properties: The JD Radius of Gyration (RG), and the hairpin angle, successfully employed in recent literature to describe the sampling of JD conformations [12,38].

## Supplementary Materials

Supplementary materials can be found at http://www.mdpi.com/1422-0067/19/8/2151/s1.
