# Supplementary material for: Protein Environment: A Crucial Triggering Factor in Josephin Domain Aggregation: The Role of 2,2,2-Trifluoroethanol
Source: Int J Mol Sci. 2018 Jul 24;19(8):2151. doi: 10.3390/ijms19082151 (PMC6121581; doi:10.3390/ijms19082151)
Supplement: Supplementary file 1 [file ijms-19-02151-s001.zip › ijms-330881-suppl.pdf]

Supplementary File

Table S1. Secondary structure of fresh and aggregated JD in the presence of different TFE concentrations. Assignment of secondary structures of ATR/FTIR spectra acquired after 0 and 24 h of incubation at 37 °C in the presence of 0, 1, 2 and 5% TFE. All the deconvolutions were performed using PickFit software.

		JD + 0% TFE		JD + 1% TFE		JD + 2% TFE		JD + 5% TFE	
		t 0	t 24	t 0	t 24	t 0	t 24	t 0	t 24
		Area (%)	Area (%)	Area (%)	Area (%)	Area (%)	Area (%)	Area (%)	Area (%)
**1624 cm^{-1}**	**random coil**	6.8	-	5.7	-	6.3	-	7.8	-
**1628 cm^{-1}**	**ß-sheet (inter)**	-	32.1	-	35.3	-	36.6	-	40.4
**1635 cm^{-1}**	**ß-sheet (intra)**	34.1		34.3	-	35.7	-	33.6	-
**1657 cm^{-1}**	**α-helix**	44.0	41.1	45.3	38.7	45.7	35.4	45.9	41.4
**1672/1681 cm^{-1}**	**turns**	15.1	20.2	14.7	19.7	12.3	19.8	12.7	18.5
**1687 cm^{-1}**	**ß-sheet (inter)**	-	6.6	-	6.3	-	8.2	-	-
